# Serum exosomal coronin 1A and dynamin 2 as neural tube defect biomarkers

**DOI:** 10.1007/s00109-022-02236-w

**Published:** 2022-08-01

**Authors:** Yanfu Wang, Ling Ma, Shanshan Jia, Dan Liu, Hui Gu, Xiaowei Wei, Wei Ma, Wenting Luo, Yuzuo Bai, Weilin Wang, Zhengwei Yuan

**Affiliations:** 1 Department of Pediatric Surgery, Key Laboratory of Health Ministry for Congenital Malformation, Shengjing Hospital, China Medical University, No. 36, Sanhao Street, Heping District, Shenyang, People’s Republic of China; 2 Department of Pediatric Surgery, Neonatal Surgery, Shengjing Hospital, China Medical University, Shenyang, People’s Republic of China; 3 Department of Pathophysiology, College of Basic Medical Science, China Medical University, Shenyang, People’s Republic of China

**Keywords:** Neural tube defects, Spina bifida aperta, Prenatal diagnosis, Proteomics, Exosome

## Abstract

**Abstract:**

No highly specific and sensitive biomarkers have been identified for early diagnosis of neural tube defects (NTDs). In this study, we used proteomics to identify novel proteins specific for NTDs. Our findings revealed three proteins showing differential expression during fetal development. In a rat model of NTDs, we used western blotting to quantify proteins in maternal serum exosomes on gestational days E18, E16, E14, and E12, in serum on E18 and E12, in neural tubes on E18 and E12, and in fetal neural exosomes on E18. The expression of coronin 1A and dynamin 2 was exosome-specific and associated with spina bifida aperta embryogenesis. Furthermore, coronin 1A and dynamin 2 were significantly downregulated in maternal serum exosomes (E12–E18), neural tubes, and fetal neural exosomes. Although downregulation was also observed in serum, the difference was not significant. Differentially expressed proteins were further analyzed in the serum exosomes of pregnant women during gestational weeks 12–40 using enzyme-linked immunosorbent assays. The findings revealed that coronin 1A and dynamin 2 showed potential diagnostic efficacy during gestational weeks 12–40, particularly during early gestation (12–18 weeks). Therefore, these two targets are used as candidate NTD screening and diagnostic biomarkers during early gestation.

**Key messages:**

- We used proteomics to identify novel proteins specific for NTDs.
- CORO1A and DNM2 showed exosome-specific expression and were associated with SBA.
- CORO1A and DNM2 were downregulated in maternal serum exosomes and FNEs.
- CORO1A and DNM2 showed good diagnostic efficacy for NTDs during early gestation.
- These two targets may have applications as NTD screening and diagnostic biomarkers.

**Supplementary information:**

The online version contains supplementary material available at 10.1007/s00109-022-02236-w.

## Introduction

Neural tube defects (NTDs) are common congenital malformations that cause severe damage to the fetus as a result of the failure of neural tube closure at 21–28 days after conception [1]. Spina bifida aperta (SBA) is one of the most common types of NTDs and presents with urinary and neurological complications [2]. Moreover, after surgery, long-term follow-up therapy and medical support may be necessary [3].

Ultrasound is one of the most powerful diagnostic tools for NTDs [4]; however, this approach is less effective for identifying NTDs in low-risk pregnancies, particularly during the first trimester [5], because NTDs can only be detected after defect formation is complete. The American Congress of Obstetricians and Gynecologists recommends maternal serum alpha-fetoprotein (MSAFP) screening and specialized ultrasound examination to be offered to all pregnant women and those found at high risk for NTDs to identify the defect [6]. Unfortunately, the MSAFP detection rate is only approximately 65–80% when using a cutoff of 2.5 multiples of the median because of various factors [7]. Elevated MSAFP can also be caused by conditions such as fetal abdominal wall defects, congenital kidney disease, fetal death, placenta accreta, preeclampsia, and oligohydramnios [8, 9]. We previously combined the analysis of serum complements (including complement C1q A chain, complement C1s, and complement C3) and MSAFP to overcome the shortcomings of MSAFP analysis and improve the sensitivity and specificity of NTD screening [10]. Indeed, the identification of prenatal molecular biomarkers, particularly specific and sensitive maternal serum biomarkers, may facilitate the early screening, diagnosis, and treatment of NTDs during early gestation.

Extracellular vesicles (EVs) can pack and preserve proteins, RNA, and DNA, enrich and transfer informative factors with more specificity, and are not easily degraded in bodily fluids [11]. In particular, exosomes permit the specific transport of cargo to target cells during pregnancy [12] and have been shown to be more stable than the other types of EVs [13]. Exosomes can be detected at high levels during gestational week 6 [14], and the contents of these EVs are altered under pathological conditions [15]. Exosomes have been found in the placenta [16] and amniotic fluid [17] and can transfer to the maternal side [18]. Indeed, approximately 35% of total maternal exosomes are fetal [19]. Furthermore, fetal neural exosomes (FNEs) change when exposed to ethyl alcohol [20, 21]. Therefore, exosomes may carry specific molecular biomarkers from the fetus to the mother and may have potential applications in prenatal screening and diagnosis.

Proteomics is a powerful tool that can identify specific biomarkers of NTDs and their underlying molecular mechanisms in relevant animal models and clinical samples [22]. We previously found 14–3-3ζ present distinctly in fetal neural tubes [23], apolipoprotein A4 and alpha-fetoprotein (AFP) fragment changes in amniotic fluid [24], and proprotein convertase subtilisin/kexin type 9 changes in serum [25]. However, no differentially expressed exosomal proteins have been identified as diagnostic biomarkers of NTDs.

Accordingly, in this study, we used proteomics and bioinformatics to screen for enriched proteins in maternal serum exosomes as potential SBA biomarkers.

## Materials and methods

### Clinical sample collection

Serum samples from pregnant women were collected from the biological specimen bank of the Shengjing Birth Cohort in Key Laboratory of Health Ministry for Congenital Malformation, Shenyang, China. Nineteen pregnant women were diagnosed as carrying fetuses with NTDs using prenatal ultrasound, and the diagnosis was confirmed by autopsy after induced labor or by physical examination after delivery. Serum samples from gestational age- and maternal age-matched controls were obtained from pregnant women (*n* = 19) carrying normal fetuses without any abnormalities. Every pregnant woman consumed folic acid regularly under guidance during pregnancy. The clinical characteristics of the patients are summarized in Online Resource 1.

### Animal sample collection

SBA was induced in Wistar rats with all-trans-retinoic acid (atRA; Sigma, St. Louis, MO, USA; 4% [w/v] in olive oil; 140 mg/kg body weight) at E10 (vaginal smear containing sperm designated E0) by gavage, and normal controls were treated with the same volume of oil, as described previously [24, 26]. Briefly, animals were anesthetized with isoflurane, and blood was collected from the apex cordis of living rats; euthanasia was then carried out by CO_2_ asphyxiation. All experimental protocols involving animals were approved by the Medical Ethics Committee of the Shengjing Hospital of China Medical University (2016PS106K).

**Fig. 1:**
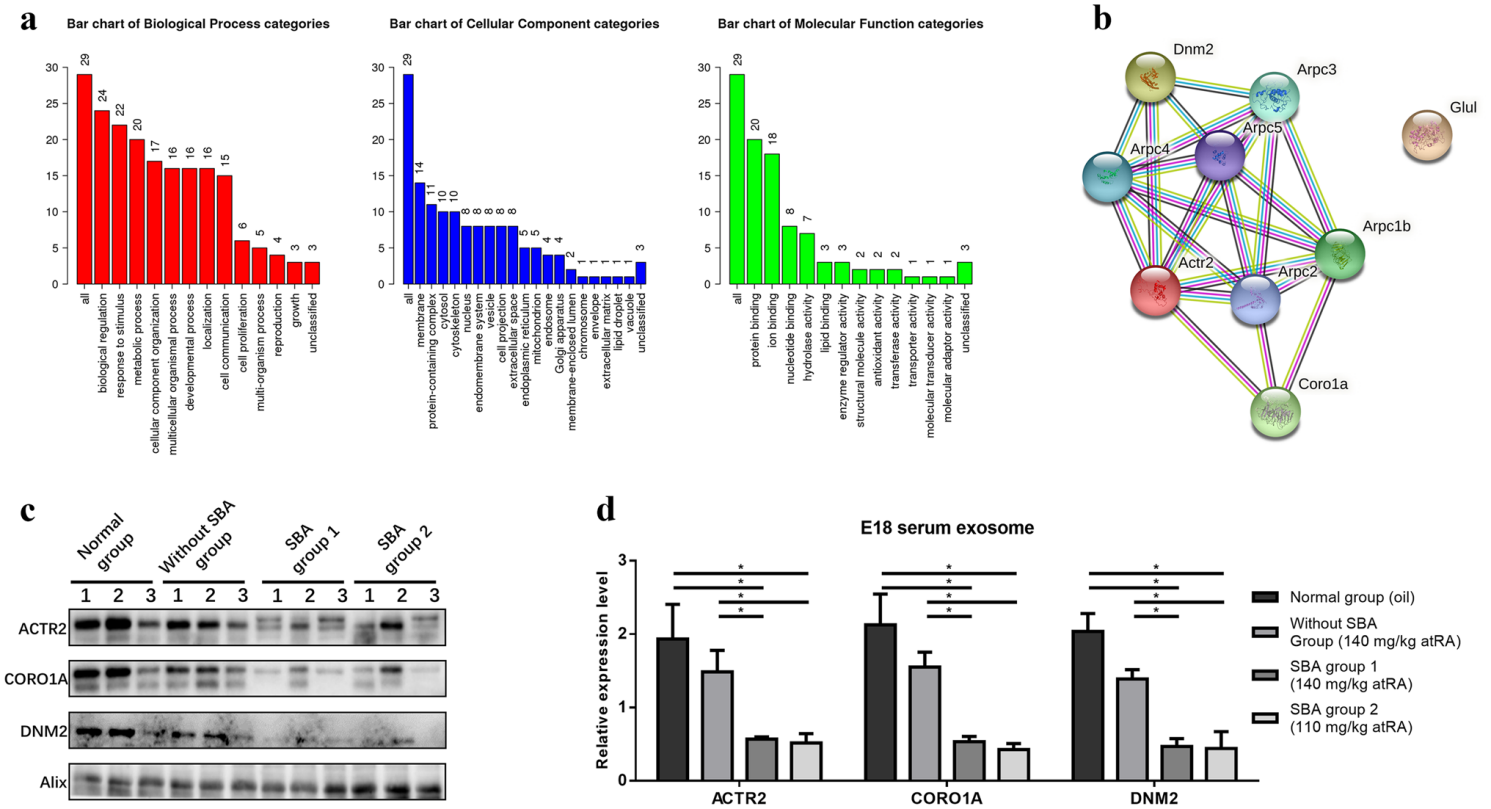
Bioinformatics and comparative analyses in serum exosomes of pregnant rats at E18. **a** Bar chart of gene ontology (GO) biological process, cellular component, and molecular function categories. **b** Protein network of ACTR2, CORO1A, and DNM2. **c** Western blot analysis of ACTR2, CORO1A, and DNM2 expressions in serum exosomes at E18 among normal group (treated with oil, *n* = 3), without SBA group (treated with 140 mg/mL atRA, *n* = 3), SBA group 1 (treated with 140 mg/mL atRA, *n* = 3), and SBA group 2 (treated with 110 mg/mL atRA, *n* = 3). **d** Bar chart of the relative expression of ACTR2, CORO1A, and DNM2 among the four groups in serum exosomes at E18, as determined by western blotting

Fetal deformities were examined using stereomicroscopy (M165 FC; Leica, Mannheim, Germany; Online Resource 4. Fig. 1). Blood samples were collected into vacuum tubes (Vacutainer SST; Becton, Dickinson and Company, Franklin Lake, NJ, USA) and centrifuged (Sorvall ST8R Centrifuge; Thermo Fisher Scientific, Walther, MA, USA) at 2000 × *g* and 4 °C for 20 min for serum sampling. Serum samples were collected from normal pregnant rats with normal embryos at E12 (*n* = 12), E14 (*n* = 6), E16 (*n* = 6), and E18 (*n* = 25) and from pregnant rats with SBA embryos at E12 (*n* = 12), E14 (*n* = 6), E16 (*n* = 6), and E18 (*n* = 25). We also collected 12 treated E18 samples from normal (*n* = 3) rats treated with oil, those without SBA (*n* = 3) from rats treated with 140 mg/kg atRA, those with SBA (*n* = 3) from rats treated with 140 mg/kg atRA (SBA group 1), and those with SBA (*n* = 3) from rats treated with 110 mg/kg atRA (SBA group 2; Online Resource 2). The fetal neural tube tissue (E12) or spinal cord tissue (E18) (from the inferior margin of the forelimb bud to the tail bud) was isolated in cold phosphate-buffered saline (PBS). Samples were stored at − 80 °C, except tissues used for immunohistochemistry, which were preserved in 4% paraformaldehyde and then embedded in paraffin.

### Total serum exosome extraction and characterization

**Fig. 2:**
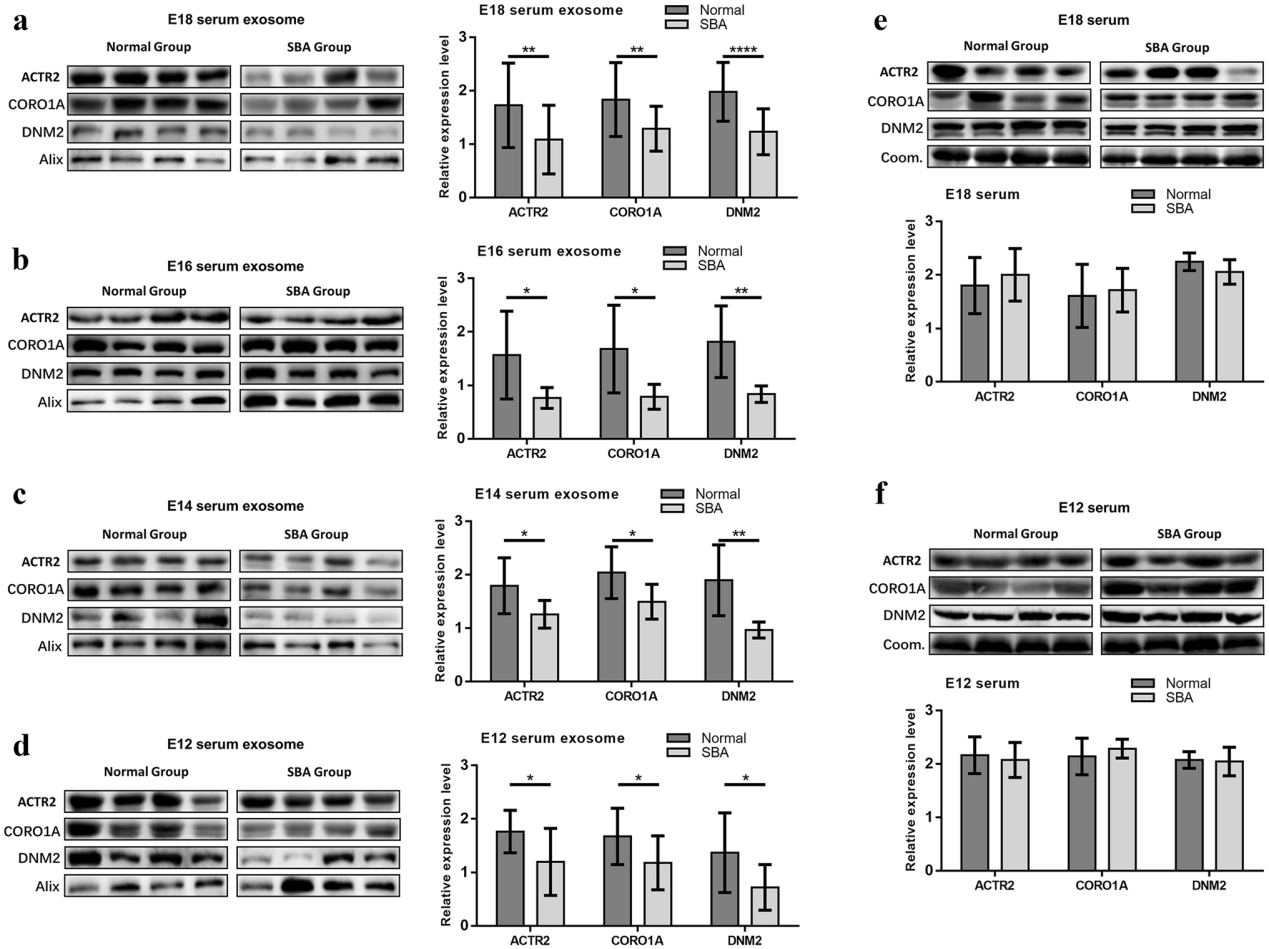
Analyses of ACTR2, CORO1A, and DNM2 expressions in serum exosomes and serum without isolation of exosomes of pregnant rats. **a** Western blot analysis of protein expression in serum exosomes at E18 (complete bands are shown in Online Resource 4. Fig. 3); bar chart of relative protein expression at E18 in the normal (*n* = 19) and SBA (*n* = 19) groups. **b** Western blot analysis of protein expression in serum exosomes at E16 (complete bands are shown in Online Resource 4. Fig. 4); bar chart of relative protein expression at E16 in the normal (*n* = 6) and SBA (*n* = 6) groups. **c** Western blot analysis of protein expression in serum exosomes at E14 (complete bands are shown in Online Resource 4. Fig. 5); bar chart of relative protein expression at E14 in the normal (*n* = 6) and SBA (*n* = 6) groups. **d** Western blot analysis of protein expression in serum exosomes at E12 (complete bands are shown in Online Resource 4. Fig. 6); bar chart of relative protein expression at E12 in the normal (*n* = 12) and SBA (*n* = 12) groups. **e** Western blot analysis of protein expression in serum without isolation of exosomes at E18 (complete bands are shown in Online Resource 4. Fig. 7); bar chart of relative protein expression in serum without isolation of exosomes at E18 in the normal (*n* = 6) and SBA (*n* = 6) groups. **f** Western blot analysis of protein expression in serum without isolation of exosomes at E12 (complete bands are shown in Online Resource 4. Fig. 8); bar chart of relative protein expression in serum without isolation of exosomes at E12 in the normal (*n* = 6) and SBA (*n* = 6) groups

A 200-μL aliquot of each serum sample was adjusted to 3 mL with phosphate-buffered saline (PBS) and utilized for extraction. The diluted serum was filtered through a 0.22-μm filter (MILLEX GP, Millipore Express PES Membrane; Millipore, Billerica, MA, USA) [27]. The filtered sample was centrifuged (Micro Ultracentrifuge CS120FNX; Hitachi Koki, Tokyo, Japan) at 10,000 × *g* and 4 °C for 1 h. Subsequently, the supernatant was transferred to a fresh tube and centrifuged at 100,000 × *g* and 4 °C for 4 h. The pellets were washed with PBS, ultracentrifuged at 100,000 × *g* and 4 °C for 1 h again to purify the exosomes [28]. The pellet was resuspended in 100 µL PBS and preserved at − 80 °C as previously described [29, 30]. Characterization of the extracted exosomes was confirmed by transmission electron microscopy (HT7800; Hitachi Koki, Tokyo, Japan), dynamic light scattering (Nano ZS90; Malvern Instruments, UK), and exosomal biomarkers (Alix, CD63, and CD9; Online Resource 4. Fig. 2).

### FNE isolation

Exosomes from fetal neural sources were isolated as previously described [21, 22]. Briefly, 400 µL sample was incubated for 90 min at 20 °C with 50 µL of 3% bovine serum albumin (BSA; Solarbio, Beijing, China) containing 2 µg polyclonal goat IgG anti-rat Contactin-2/TAG1 antibody (AF4439; R&D Systems, Minneapolis, MIN, USA) that had been biotinylated (EZ-Link sulfo-NHS-biotin System; Thermo Fisher Scientific). Then, 10 µL Streptavidin-Plus UltraLink resin (Pierce; Thermo Fisher Scientific) in 40 µL of 3% BSA was added. After centrifugation, the supernatant was transferred to an Eppendorf tube and stored at − 80 °C.

### LC–MS/MS for biomarker screening

Three pairs of E18 samples were screened for potential biomarkers using label-free LC–MS/MS [31, 32]. Samples were first subjected to immunoaffinity depletion of high-abundance serum proteins. Then, 200 μg protein for each sample was processed by filter-aided sample preparation digestion. The peptide content was estimated by determining the ultraviolet spectral density at 280 nm, calculated based on the frequency of tryptophan and tyrosine in vertebrate proteins [32]. Each fraction was then injected for nanoLC-MS/MS analysis. The peptide mixture was separated with a linear gradient controlled by IntelliFlow technology. LC–MS/MS analysis was performed on a Q Exactive mass spectrometer (Thermo Fisher Scientific) coupled to an Easy nLC (Proxeon Biosystems). The MS data were analyzed using MaxQuant software version 1.5.3.17 (Max Planck Institute of Biochemistry, Martinsried, Germany) [33].

### Bioinformatics analysis

Bioinformatics analysis was carried out using WebGestalt (http://www.webgestalt.org/) with over-representation analysis (ORA) in *Rattus norvegicus* for enrichment with the gene ontology (GO) biological process functional database. The significance level in the advanced parameters was adjusted to a false-discovery rate of less than 0.05. The proteins showing enrichment were further analyzed using the String database (https://string-db.org/) to define protein interaction networks.

### Western blotting

Exosome-derived from serum (E12, E14, E16, E18), serum (E12, E18), neural tube tissue (E12), and spinal cord tissue (E18) were treated with 20 µL radioimmunoprecipitation assay buffer (Solarbio) per 100 µL sample and subjected to ultrasound pyrolysis. The supernatants were collected as the protein-containing fraction. Serum (E12, E18) was diluted by PBS before western blotting. Samples were quantified using a BCA Protein Assay Kit (Solarbio), and protein concentrations were adjusted using PBS. The diluted samples were mixed with loading buffer, denatured, separated on Bio-Rad gels, and transferred to 0.45-μm polyvinylidene difluoride membranes. The primary antibodies used were as follows: anti-Alix (3A9 mouse mAb; Cell Signaling Technology, Danvers, MA, USA; exosome internal reference), anti-CD9 (D3H4P rabbit mAb; Cell Signaling Technology), anti-CD63 (TS63 mouse mAb; Abcam, Cambridge, UK), anti-β-actin (66009–1-Ig rabbit mAb; Proteintech, Wuhan, China; neural tube internal reference), anti-actin-related protein 2 (ACTR2; 10922–1-AP rabbit pAb; Proteintech), anti-coronin 1A (CORO1A; EPR19467-36 rabbit mAb; Abcam), and anti-dynamin 2 (DNM2; EPR9053 rabbit mAb; Abcam). The secondary antibodies used were goat anti-mouse (G-21040; Invitrogen, Carlsbad, CA, USA) or goat anti-rabbit (G-21234; Invitrogen). Fast-staining Coomassie Brilliant Blue (Solarbio) served as an internal reference for serum. Bands were visualized using chemiluminescent horseradish peroxidase-based substrate (Immobilon Western; Millipore) and captured (cSeries 300; Azure Biosystems, Dublin, CA, USA). Representative samples are shown, and raw data for the bands are shown in the Online Resource 4.

### Immunohistochemistry

An UltraSensitive SP Kit (MXB Biotechnologies, Fuzhou, China) was used for immunohistochemistry, according to the manufacturer’s protocol. The primary antibodies were the same as used for western blotting. Sections were washed in PBS between each process and finally stained with diaminobenzidine and hematoxylin, dehydrated, and sealed with resin. Images were captured using a microscope (ECLIPSE 80i; Nikon, Tokyo, Japan).

#### ELISA

Serum (50 µL) was diluted with PBS (50 µL), and samples were subjected to ELISA using a human DNM2 ELISA kit (abx250599; 96-well; Abbexa) and human CORO1A ELISA kit ab214032; 96-well; Abcam), according to the manufacturer’s protocol. Absorbance was measured at 450 nm using a microplate reader (M200 PRO; Tecan, Switzerland). The relative optical density at 450 nm (OD_450_; OD_450_ of each well–OD_450_ of the blank well) was calculated.

### Statistical analysis

Data are expressed as means ± standard deviations. Western blotting bands were quantified using ImageJ (1.37c) as the ratio of the gray value of the biomarker protein/the gray value of the internal reference protein. Quantitative variables were analyzed using unpaired *t*-tests, paired *t*-tests, and one-way analysis of variance (ANOVA). Differences with *P* values less than 0.05 were considered significant. Statistical analyses were performed using GraphPad Prism 8.0 software. The diagnostic capacity of biomarkers was analyzed using receiver operating characteristic (ROC) curves, and the area under curve (AUC), specificity, and sensitivity were determined using MedCalc 19.3.1 in Statistics-ROC.

## Results

### Identification of serum exosome protein biomarkers of SBA using LC–MS/MS

Proteomics analyses revealed the presence of 397 proteins in serum exosomes (Online Resource 3), of which 33 were differentially expressed between the SBA and normal groups. There were 7 proteins specifically expressed in the SBA group and 12 proteins specifically expressed in the normal group excluding potential contaminants. Another 14 proteins were expressed in both the SBA and normal groups with a significant difference (one upregulated and 13 downregulated; Table 1). GO analysis was conducted to identify the potential functions of these proteins (excluding four uncharacterized proteins) in three categories (Fig. 1a), and ORA was used to obtain GO enrichments of biological processes with four enrichment processes (Table 2). ACTR2, CORO1A, DNM2, angiotensinogen precursor, glutamate-ammonia ligase (GLUL), LIM zinc finger domain containing 1, programmed cell death 6, and pyruvate kinase muscle isozyme were involved in more than two biological processes.

**Table 1:** List of 33 differentially expressed proteins

Protein IDs	Protein names	Gene names	Ratio
**Upregulated**			
Q6P6T1	Complement C1s subcomponent	C1s	2.99
**Downregulated**			
F1LZ11	Uncharacterized protein	N/A	0.65
Q62636	Ras-related protein Rap-1b	Rap1b	0.65
P01015	Angiotensinogen	Agt	0.63
D3ZPL2	Uncharacterized protein	N/A	0.59
P09606	Glutamine synthetase	Glul	0.55
F1M5X4	Ig-like domain-containing protein	N/A	0.55
P68136	Actin, alpha skeletal muscle	Acta1	0.54
Q8K3U6	Coagulation factor VII	F7	0.53
A0A0G2JV65	14–3-3 protein zeta/delta	Ywhaz	0.52
B0BNJ1	LOC683667 protein	Sri	0.50
A0A0G2K9Z5	Uncharacterized protein	N/A	0.48
G3V7W1	Programmed cell death protein 6	Pdcd6	0.45
P01883	Ig delta chain C region (fragment)	N/A	0.09
**Specific in normal samples**			
A0A0A0MY48	Dynamin-2	Dnm2	
Q5M7U6	Actin-related protein 2	Actr2	
Q6P502	T-complex protein 1 subunit gamma	Cct3	
G3V9N9	Alpha-1,2-mannosidase	Man1a1	
A0A0G2K393	Pleckstrin	Plek	
A0A0H2UHM5	Protein disulfide-isomerase	Pdia3	
C0KUC5	LIM and senescent cell antigen-like-containing domain protein	Lims1	
Q7M094	Destrin-like protein p17a (fragments)	Dstn	
Q5U329	Anion exchange protein	Slc4a1	
P50115	Protein S100-A8	S100a8	
Q6LC76	Fibronectin (fragment)	Fn1	
Q91ZN1	Coronin-1A	Coro1a	
**Specific in SBA samples**			
A0A0G2K2X4	Olfactory receptor	LOC100911127	
Q5I0L8	Angiopoietin-like 3	Angptl3	
M0R7M5	Uncharacterized protein	LOC100911032	
P01836	Ig kappa chain C region, A allele	N/A	
P11980	Pyruvate kinase PKM	Pkm	
Q08420	Extracellular superoxide dismutase [Cu–Zn]	Sod3	
Q91WX0	Complement factor H-related protein	RGD1564614

**Table 2:** List of gene ontology analyses of the biological processes associated with the 29 differentially expressed proteins

Gene set	Description	Size	Expect	Ratio	*P* value	FDR
GO:0008064	Regulation of actin polymerization or depolymerization	122	0.2374	21.058	3.30E − 06	0.005217
GO:0022603	Regulation of anatomical structure morphogenesis	682	1.3273	6.7805	3.19E − 06	0.005217
GO:1901700	Response to oxygen-containing compound	1542	3.0011	4.3317	1.50E − 06	0.005217
GO:0022607	Cellular component assembly	1997	3.8867	3.6021	4.34E − 06	0.00563

Among these eight proteins, ACTR2 [34, 35], CORO1A [36], GLUL [37], and DNM2 [38] function during neural tube development but have not been shown to be connected with NTDs. Analysis of the four proteins in the String database indicated that ACTR2, CORO1A, and DNM2 may be connected by the same cluster (Fig. 1b), necessitating further verification.

### Comparative analysis to exclude the effects of atRA on serum exosome biomarkers at E18

To clarify whether atRA itself could affect the expression of these biomarkers, we divided samples into four groups. ACTR2, CORO1A, and DNM2 were slightly downregulated in the non- SBA group treated with atRA compared with that in the normal group treated with oil and were significantly downregulated in SBA groups 1 and 2 compared with that in the normal group (Fig. 1c). One-way ANOVA supported this result (Fig. 1d). Thus, the decreased expression of ACTR2, CORO1A, and DNM2 in serum exosomes was related to the embryogenesis of SBA and was not a result of transcriptional repression by atRA.

### Analysis of the differential expression of ACTR2, CORO1A, and DNM2 in serum exosomes at E12–E18

Nineteen paired E18 serum exosome samples (different from those used in proteomics) were examined. ACTR2, CORO1A, and DNM2 were significantly downregulated in SBA. Unpaired *t*-tests supported these findings at E18 (ACTR2, *P* = 0.0095; CORO1A, *P* = 0.0057; DNM2, *P* < 0.0001) (Fig. 2a). To confirm the differential expression of proteins during early gestation, western blotting analysis was performed on serum exosomes at E16 (Fig. 2b), E14 (Fig. 2c), and E12 (Fig. 2d); similar trends were observed compared with the results from E18. Unpaired *t*-tests supported the significance of the findings at E16 (ACTR2, *P* = 0.0421; CORO1A, *P* = 0.0277; DNM2, *P* = 0.0059) (Fig. 2b), E14 (ACTR2, *P* = 0.0490; CORO1A, *P* = 0.0454; DNM2, *P* = 0.0073) (Fig. 2c), and E12 (ACTR2, *P* = 0.0163; CORO1A, *P* = 0.0282; DNM2, *P* = 0.0157) (Fig. 2d), similar to the results at E18. Collectively, these results suggested that these proteins exhibited lower expression in the SBA group from E12 to E18, indicating their diagnostic capacity during early gestation.

### Analysis of the differential expression of ACTR2, CORO1A, and DNM2 in serum without isolation of exosomes

To confirm the specific differential expression of these proteins in serum exosomes, we quantified their expression in whole serum without exosome isolation using western blotting. We found no obvious changes between the normal and SBA groups at E18 (Fig. 2e) and E12 (Fig. 2f), indicating their serum exosome-specific downregulation. Coomassie Brilliant Blue staining of total proteins served as an internal reference for serum (complete gel staining is shown in Online Resource 4. Figs. 7, 8). Unpaired *t*-tests showed that there were no significant differences at E18 (ACTR2, *P* = 0.5082; CORO1A, *P* = 0.7230; DNM2, *P* = 0.1316) (Fig. 2e) or E12 (ACTR2, *P* = 0.6568; CORO1A, *P* = 0.3789; DNM2, *P* = 0.8318) (Fig. 2f).

### Analysis of the differential expression of ACTR2, CORO1A, and DNM2 in fetal neural tubes and FNEs

**Fig. 3:**
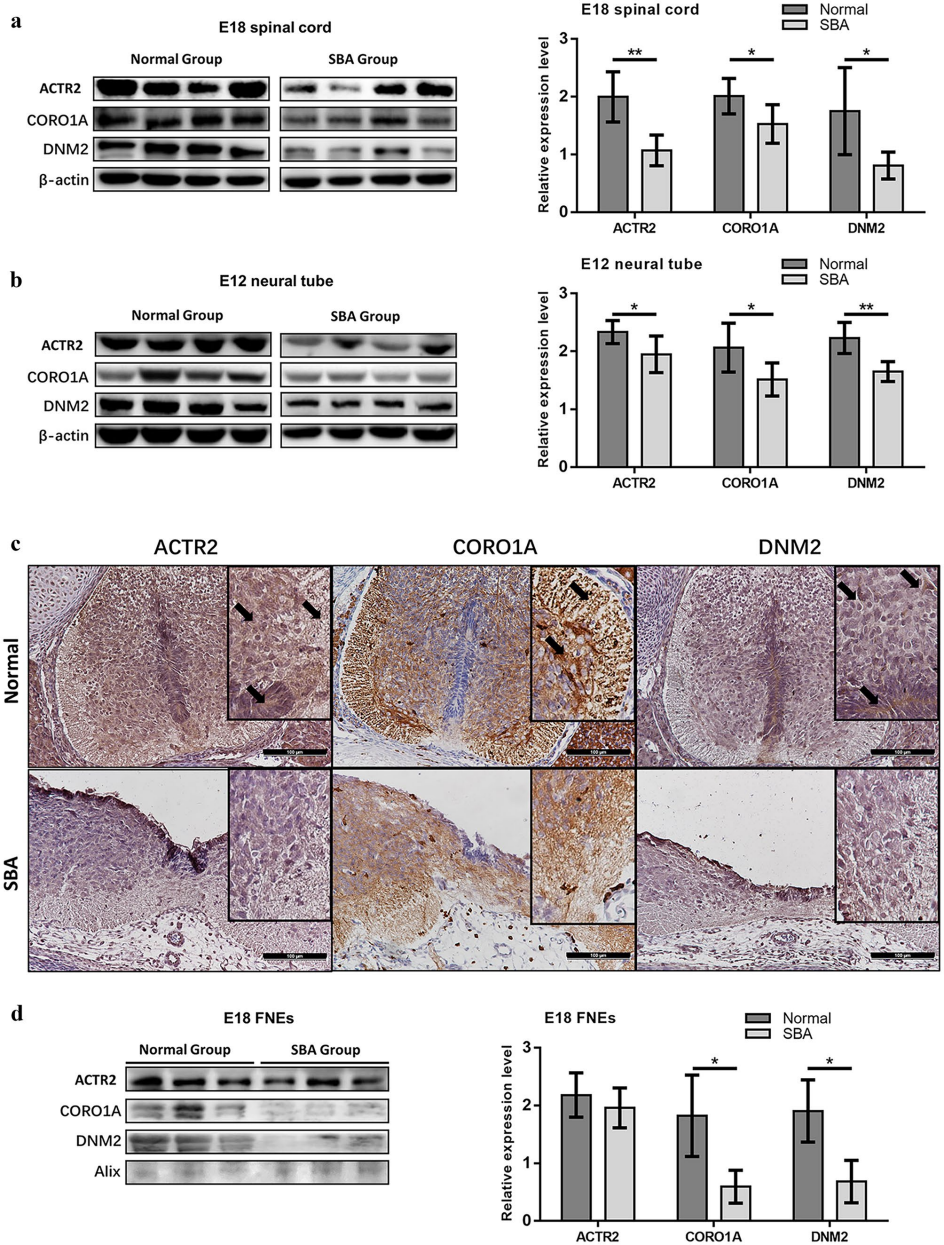
Analyses of ACTR2, CORO1A, and DNM2 expressions in spinal cords, neural tubes, and FNEs. **a** Western blot analysis of protein expression in spinal cords at E18 (complete bands are shown in Online Resource 4. Fig. 9); bar chart of relative protein expression at E18 in the normal (*n* = 6) and SBA (*n* = 6) groups. **b** Western blot analysis of protein in neural tubes at E12 (complete bands are shown in Online Resource 4. Fig. 10); bar chart of relative protein expression at E12 in the normal (*n* = 6) and SBA (*n* = 6) groups. **c** Figures above: expression of ACTR2 in the spinal cord of normal embryos, localized to the neuroepithelium (NE), neural cells (NCs), and neural fibers (NFs) (black arrow), expression of CORO1A in the spinal cord of normal embryos, localized to NFs and NCs (black arrow), expression of DNM2 in the spinal cord of normal embryos, localized to the NE and NCs (black arrow); figures below: expression of ACTR2, CORO1A, and DNM2 in the spinal cord of SBA embryos. **d** Western blot analysis of ACTR2, CORO1A, and DNM2 expressions in FNEs at E18 in the normal and SBA groups; bar chart of relative protein expression in FNEs at E18 in the normal (*n* = 3) and SBA (*n* = 3) groups, as determined by western blotting

To verify whether the downregulation of these proteins in serum exosomes was related to NTDs, we quantified their expression in SBA spinal cords at E18 (Fig. 3a) and neural tubes at E12 (Fig. 3b) using western blotting. The expression patterns were the same as in serum exosomes, showing significantly decreased expression in SBA. Unpaired *t*-tests supported these findings at E18 (ACTR2, *P* = 0.0012; CORO1A, *P* = 0.0264; DNM2, *P* = 0.0151) (Fig. 3a) and E12 (ACTR2, *P* = 0.0301; CORO1A, *P* = 0.0247; DNM2, *P* = 0.0012) (Fig. 3b).

Immunohistochemistry was performed in E18 embryos to evaluate the localization and expression levels of these proteins in neural tubes. ACTR2 was expressed in all cells within the field of vision, with upregulation in the neuroepithelium, neural cells, and neural fibers in normal control and downregulation in SBA. CORO1A was upregulated in nerve fibers in normal control but downregulated in SBA. DNM2 was upregulated in the neuroepithelium and neural cells in normal control but hardly detected in SBA (Fig. 3c).

To further verify whether ACTR2, CORO1A, and DNM2 downregulated in total serum exosomes originated from the fetal neural source, we tested the proteins in FNEs isolated from total serum exosomes of maternal rats. Changes in CORO1A and DNM2 expressions in FNEs followed the same pattern as in total serum exosomes, with downregulation in SBA (CORO1A, *P* = 0.0487; DNM2, *P* = 0.0316); ACTR2 expression did not differ between normal samples and SBA FNEs samples (ACTR2, *P* = 0.4987) (Fig. 3d). Thus, CORO1A and DNM2 expressions were specific to neural cells and tissues.

### Analysis of the differential expression of CORO1A and DNM2 in serum exosomes from pregnant women

**Fig. 4:**
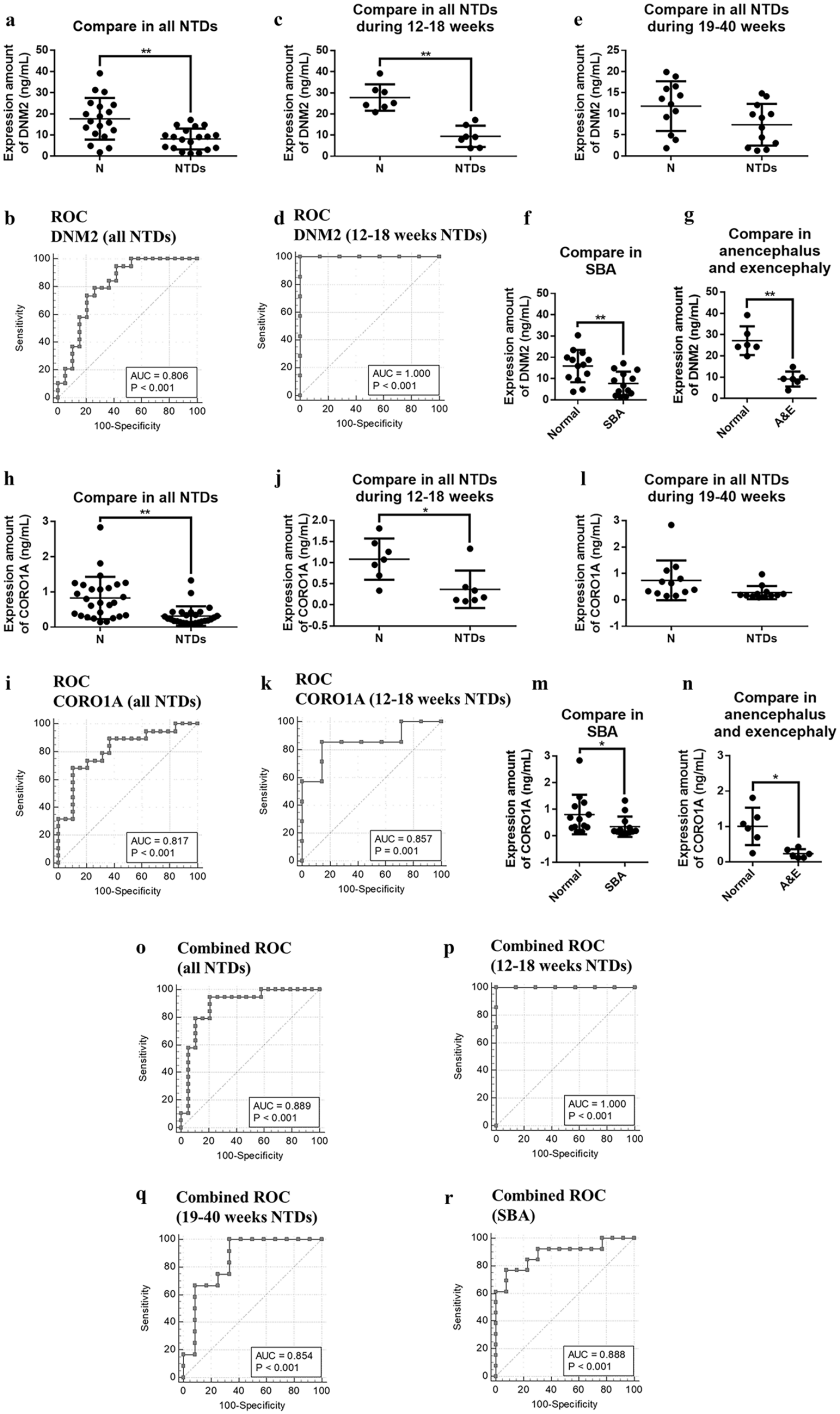
Analyses of biomarker expression in serum exosomes of pregnant women. **a** Scatter diagram of ELISA for DNM2 expression in serum exosomes during gestational weeks 12–40, including 19 normal samples and 19 NTD samples. **b** ROC curves for DNM2 are shown. **c** Scatter diagram of ELISA for DNM2 and expression in serum exosomes during gestational weeks 12–18, including 7 normal samples and 7 NTD samples. **d** ROC curves for DNM2 are shown. **e** Scatter diagram of ELISA for DNM2 expression in serum exosomes during gestational weeks 19–40, including 12 normal samples and 12 NTD samples. Scatter diagram of ELISA in serum exosomes among **f** 13 normal samples and 13 SBA samples for DNM2 and **g** 6 normal samples and 6 anencephalus and exencephalus samples for DNM2. **h** Scatter diagram of ELISA for CORO1A expression in serum exosomes during gestational weeks 12–40, including 19 normal samples and 19 NTD samples. **i** ROC curves for CORO1A are shown. **j** Scatter diagram of ELISA for CORO1A and expression in serum exosomes during gestational weeks 12–18, including seven normal samples and seven NTD samples. **k** ROC curves for CORO1A are shown. **l** Scatter diagram of ELISA for CORO1A expression in serum exosomes during gestational weeks 19–40, including 12 normal samples and 12 NTD samples. Scatter diagram of ELISA in serum exosomes among **m** 13 normal samples and 13 SBA samples for CORO1A and **n** 6 normal samples and 6 anencephalus and exencephalus samples for CORO1A. Combined ROC curves for CORO1A and DNM2 expressions in NTD samples during **o** gestational weeks 12–40, **p** gestational weeks 12–18, and **q** gestational weeks 19–40 and in SBA samples during **r** gestational weeks 12–40

Next, we performed ELISA of DNM2 and CORO1A in maternal serum exosomes to validate their expression. Samples from pregnant women were paired according to gestational week, and paired *t*-tests were used for statistical analysis. Compared with normal controls, all 19 pregnant women who were diagnosed as carrying fetuses with NTDs showed a significant decrease in DNM2 expression (*P* = 0.0011) (Fig. 4a), and the ROC curve showed high accuracy (specificity, 78.95%; sensitivity, 73.68%; AUC, 0.806) (Fig. 4b). Analysis during different pregnancy periods showed that DNM2 was significantly downregulated at gestational weeks 12–18 (*P* = 0.0034) (Fig. 4c), and this marker showed extremely high accuracy (specificity, 100%; sensitivity, 100%; AUC, 1.000) (Fig. 4d). Downregulation was not significant at gestational weeks 19–40 (*P* = 0.0557) (Fig. 4e). Subgroup analysis showed significant differences in DNM2 expression in the SBA group (*P* = 0.0035) (Fig. 4f) and the anencephalus and exencephalus group (*P* = 0.0058) (Fig. 4g).

CORO1A expression was also significantly downregulated in the 19 pregnant women compared with that in normal controls (*P* = 0.0022) (Fig. 4h), and its ROC curve showed high accuracy (specificity, 89.47%; sensitivity, 68.42%; AUC, 0.817) (Fig. 4i). Furthermore, significant downregulation was observed at gestational weeks 12–18 (*P* = 0.0108) (Fig. 4j), and this marker showed high accuracy (specificity, 85.71%; sensitivity, 85.71%; AUC, 0.857) (Fig. 4k). No significant downregulation was observed at gestational weeks 19–40 (*P* = 0.0599) (Fig. 4l). Subgroup analysis showed that CORO1A was differentially expressed in the SBA group (*P* = 0.0440) (Fig. 4m) and the anencephalus and exencephalus group (*P* = 0.0186) (Fig. 4n).

We further analyzed the combined performance of DNM2 and CORO1A in diagnostic efficacy of NTDs. The ROC curve showed better performance in all 19 pair samples and exhibited higher accuracy (specificity, 78.95%; sensitivity, 94.74%; AUC, 0.889) (Fig. 4o). At gestational weeks 12–18, ROC curves showed better performance and high accuracy (specificity, 100%; sensitivity, 100%; AUC, 1.000) (Fig. 4p). At gestational weeks 19–40 (n = 12), ROC curves showed good performance (specificity, 66.67%; sensitivity, 100%; AUC, 0.854) (Fig. 4q). ROC curves also showed good performance in 13 paired SBA samples, with high accuracy and specificity (specificity, 92.31%; sensitivity, 76.92%; AUC, 0.888) (Fig. 4r).

## Discussion

In this study, we showed that ACTR2, CORO1A, and DNM2 were downregulated in serum exosomes in a SBA rat model using LC–MS/MS (label-free) in conjunction with bioinformatics analyses. CORO1A and DNM2 were also differentially expressed in FNEs, and these findings were validated in pregnant women, indicating that these two proteins may be candidate specific diagnostic biomarkers of NTDs during early gestation.

All three of these proteins have crucial roles in fetal development. CORO1A is involved in axon guidance and branching to final targets by mediating actin assembly and reorganization with cofilin and the ARP2/3 complex (ARPC3) [36]. CORO1A also stabilizes [39] and binds to [40] F-actin. The large GTPase DNM2 controls the spreading and motility of the growth cone [38] and can regulate hormone secretion and vesicle release from neuroendocrine cells [41]. F-actin requires DNM2 to polymerize and assemble with ARPC3 [42]. Moreover, reorganization of F-actin requires the DNM2/cortactin/ARPC3 complex [43]. ACTR2, a component of ARPC3, is essential during different phases of neural development, including neurogenesis, neuritogenesis, and neural migration [44]. ARPC3 is also critical for the regulation and dynamics of F-actin [35, 45, 46], which is involved in axon and dendrite growth [47, 48] as well as neural tube closure [49, 50]. Therefore, F-actin may be an effective indicator of neural development associated with these molecules.

There were some limitations to our study. First, the routine administration of folic acid during gestation has substantially reduced the occurrence of NTDs; therefore, the sample size of pregnant women carrying fetuses with NTDs was quite small. More data from clinical samples are required to confirm our findings and perform combined analysis of the potential biomarkers. Additionally, the earliest samples of NTDs were from gestational week 12, and further studies should verify whether CORO1A and DNM2 can be used to screen and diagnose NTDs before gestational week 12. Multicenter studies are also needed to validate the prenatal diagnostic potential of these biomarkers, and additional work is necessary to identify the detailed mechanisms through which CORO1A and DNM2 are related to embryogenesis in SBA.

CORO1A and DNM2 expressions in exosomes extracted from maternal serum during pregnancy may have applications in the early clinical screening and diagnosis of NTDs with high specificity. Our study established these novel and candidate molecular biomarkers and suggested their involvement in the occurrence of NTDs.

## Supplementary information

Below is the link to the electronic supplementary material.

Supplementary file1 (XLSX 12 KB)

Supplementary file2 (XLSX 11 KB)

Supplementary file3 (XLSX 161 KB)

Supplementary file4 (PDF 844 KB)

## Data Availability

The raw dataset for LC–MS/MS (label-free) is accessible on the Proteomics IDEntifications Database (https://www.ebi.ac.uk/pride/, URL: https://www.ebi.ac.uk/pride/archive/projects/PXD023775/private, project ID: PXD023775).

## References

[1] Botto LD, Moore CA, Khoury MJ, Erickson JD (1999). Neural-tube defects. N Engl J Med.

[2] Peyronnet B, Gao F, Brochard C, Oger E, Scailteux LM, Balusson F, Hascoet J, Alimi Q, Khene ZE, Bayat S et al (2019) Urological disorders are still the leading cause of inhospital death in patients with spina bifida. Urology. 10.1016/j.urology.2019.11.006 10.1016/j.urology.2019.11.006 31734348

[3] Phillips LA, Burton JM, Evans SH (2017). Spina bifida management. Curr Probl Pediatr Adolesc Health Care.

[4] Norem CT, Schoen EJ, Walton DL, Krieger RC, O’Keefe J, To TT, Ray GT (2005). Routine ultrasonography compared with maternal serum alpha-fetoprotein for neural tube defect screening. Obstet Gynecol.

[5] Krantz D, Hallahan T, Janik D, Carmichael J (2014). Maternal serum screening markers and adverse outcome: a new perspective. J Clin Med.

[6] Cheschier N, Bulletins-Obstetrics ACoP (2003) ACOG practice bulletin. Neural tube defects. Number 44, July 2003. (Replaces committee opinion number 252, March 2001). Int J Gynaecol Obstet 83:123–133. 10.1016/s0020-7292(03)00390-4 10.1016/s0020-7292(03)00390-4 14626221

[7] Palomaki GE, Bupp C, Gregg AR, Norton ME, Oglesbee D, Best RG, Committee ABGSotLQA (2019) Laboratory screening and diagnosis of open neural tube defects, 2019 revision: a technical standard of the American College of Medical Genetics and Genomics (ACMG). Genet Med. 10.1038/s41436-019-0681-0 10.1038/s41436-019-0681-0 31700163

[8] Krantz DA, Hallahan TW, Sherwin JE (2010). Screening for open neural tube defects. Clin Lab Med.

[9] Milunsky A, Jick SS, Bruell CL, MacLaughlin DS, Tsung YK, Jick H, Rothman KJ, Willett W (1989). Predictive values, relative risks, and overall benefits of high and low maternal serum alpha-fetoprotein screening in singleton pregnancies: new epidemiologic data. Am J Obstet Gynecol.

[10] Dong N, Gu H, Liu D, Wei X, Ma W, Ma L, Liu Y, Wang Y, Jia S, Huang J (2020). Complement factors and alpha-fetoprotein as biomarkers for noninvasive prenatal diagnosis of neural tube defects. Ann N Y Acad Sci.

[11] Kim DK, Kang B, Kim OY, Choi DS, Lee J, Kim SR, Go G, Yoon YJ, Kim JH, Jang SC et al (2013) EVpedia: an integrated database of high-throughput data for systemic analyses of extracellular vesicles. J Extracell Vesicles 2. 10.3402/jev.v2i0.20384 10.3402/jev.v2i0.20384 PMC3760654 24009897

[12] Akers JC, Gonda D, Kim R, Carter BS, Chen CC (2013). Biogenesis of extracellular vesicles (EV): exosomes, microvesicles, retrovirus-like vesicles, and apoptotic bodies. J Neurooncol.

[13] Panigrahi GK, Deep G (2017). Exosomes-based biomarker discovery for diagnosis and prognosis of prostate cancer. Front Biosci (Landmark Ed).

[14] Salomon C, Nuzhat Z, Dixon CL, Menon R (2018). Placental exosomes during gestation: liquid biopsies carrying signals for the regulation of human parturition. Curr Pharm Des.

[15] Arenaccio C, Federico M (2017). The multifaceted functions of exosomes in health and disease: an overview. Adv Exp Med Biol.

[16] Salomon C, Torres MJ, Kobayashi M, Scholz-Romero K, Sobrevia L, Dobierzewska A, Illanes SE, Mitchell MD, Rice GE (2014). A gestational profile of placental exosomes in maternal plasma and their effects on endothelial cell migration. PLoS ONE.

[17] Mincheva-Nilsson L, Baranov V (2010). The role of placental exosomes in reproduction. Am J Reprod Immunol.

[18] Kumar P, Becker JC, Gao K, Carney RP, Lankford L, Keller BA, Herout K, Lam KS, Farmer DL, Wang A (2019). Neuroprotective effect of placenta-derived mesenchymal stromal cells: role of exosomes. FASEB J.

[19] Sheller-Miller S, Choi K, Choi C, Menon R (2019) Cyclic-recombinase-reporter mouse model to determine exosome communication and function during pregnancy. Am J Obstet Gynecol 221: 502 e501–502 e512. 10.1016/j.ajog.2019.06.010 10.1016/j.ajog.2019.06.010 31207235

[20] Sheller-Miller S, Lei J, Saade G, Salomon C, Burd I, Menon R (2016). Feto-maternal trafficking of exosomes in murine pregnancy models. Front Pharmacol.

[21] Goetzl L, Darbinian N, Goetzl EJ (2016). Novel window on early human neurodevelopment via fetal exosomes in maternal blood. Ann Clin Transl Neurol.

[22] Goetzl L, Darbinian N, Merabova N (2019). Noninvasive assessment of fetal central nervous system insult: potential application to prenatal diagnosis. Prenat Diagn.

[23] Fan Y, Wang L, Zhou F, Zhang Y, Li H, Shan L, Yuan Z (2011). Comparative proteomics of spinal cords of rat fetuses with spina bifida aperta. J Proteomics.

[24] Shan L, Fan Y, Li H, Liu W, Gu H, Zhou F, Yuan Z (2012). Proteomic analysis of amniotic fluid of pregnant rats with spina bifida aperta. J Proteomics.

[25] An D, Wei X, Li H, Gu H, Huang T, Zhao G, Liu B, Wang W, Chen L, Ma W (2015). Identification of PCSK9 as a novel serum biomarker for the prenatal diagnosis of neural tube defects using iTRAQ quantitative proteomics. Sci Rep.

[26] Wu LN, Wei XW, Fan Y, Miao JN, Wang LL, Zhang Y, Wu D, Yuan ZW (2013). Altered expression of 14–3-3zeta protein in spinal cords of rat fetuses with spina bifida aperta. PLoS ONE.

[27] Iwai K, Minamisawa T, Suga K, Yajima Y, Shiba K (2016). Isolation of human salivary extracellular vesicles by iodixanol density gradient ultracentrifugation and their characterizations. J Extracell Vesicles.

[28] Thery C, Amigorena S, Raposo G, Clayton A (2006) Isolation and characterization of exosomes from cell culture supernatants and biological fluids. Curr Protoc Cell Biol Chapter 3: Unit 3 22. 10.1002/0471143030.cb0322s30 10.1002/0471143030.cb0322s30 18228490

[29] Jia S, Zhang Q, Wang Y, Wang Y, Liu D, He Y, Wei X, Gu H, Ma W, Luo W (2021). PIWI-interacting RNA sequencing profiles in maternal plasma-derived exosomes reveal novel non-invasive prenatal biomarkers for the early diagnosis of nonsyndromic cleft lip and palate. EBioMedicine.

[30] Ma L, Wei X, Ma W, Liu Y, Wang Y, He Y, Jia S, Wang Y, Luo W, Liu D et al (2022) Neural stem cell-derived exosomal netrin1 contributes to neuron differentiation of mesenchymal stem cells in therapy of spinal bifida aperta. Stem Cells Transl Med. 10.1093/stcltm/szac009 10.1093/stcltm/szac009 PMC9154334 35325230

[31] Jorrin-Novo JV (2014). Plant proteomics methods and protocols. Methods Mol Biol.

[32] Wisniewski JR, Zougman A, Nagaraj N, Mann M (2009). Universal sample preparation method for proteome analysis. Nat Methods.

[33] Cox J, Mann M (2008). MaxQuant enables high peptide identification rates, individualized p.p.b.-range mass accuracies and proteome-wide protein quantification. Nat Biotechnol.

[34] San Miguel-Ruiz JE, Letourneau PC (2014). The role of Arp2/3 in growth cone actin dynamics and guidance is substrate dependent. J Neurosci.

[35] Wegner AM, Nebhan CA, Hu L, Majumdar D, Meier KM, Weaver AM, Webb DJ (2008). N-wasp and the arp2/3 complex are critical regulators of actin in the development of dendritic spines and synapses. J Biol Chem.

[36] Martorella M, Barford K, Winkler B, Deppmann CD (2017). Emergent role of coronin-1a in neuronal signaling. Vitam Horm.

[37] Zhou Y, Dhaher R, Parent M, Hu QX, Hassel B, Yee SP, Hyder F, Gruenbaum SE, Eid T, Danbolt NC (2019). Selective deletion of glutamine synthetase in the mouse cerebral cortex induces glial dysfunction and vascular impairment that precede epilepsy and neurodegeneration. Neurochem Int.

[38] Kurklinsky S, Chen J, McNiven MA (2011). Growth cone morphology and spreading are regulated by a dynamin-cortactin complex at point contacts in hippocampal neurons. J Neurochem.

[39] Galkin VE, Orlova A, Brieher W, Kueh HY, Mitchison TJ, Egelman EH (2008). Coronin-1A stabilizes F-actin by bridging adjacent actin protomers and stapling opposite strands of the actin filament. J Mol Biol.

[40] Chan KT, Roadcap DW, Holoweckyj N, Bear JE (2012). Coronin 1C harbours a second actin-binding site that confers co-operative binding to F-actin. Biochem J.

[41] Yang Z, Li H, Chai Z, Fullerton MJ, Cao Y, Toh BH, Funder JW, Liu JP (2001). Dynamin II regulates hormone secretion in neuroendocrine cells. J Biol Chem.

[42] Unsworth KE, Mazurkiewicz P, Senf F, Zettl M, McNiven M, Way M, Holden DW (2007). Dynamin is required for F-actin assembly and pedestal formation by enteropathogenic Escherichia coli (EPEC). Cell Microbiol.

[43] Krueger EW, Orth JD, Cao H, McNiven MA (2003). A dynamin-cortactin-Arp2/3 complex mediates actin reorganization in growth factor-stimulated cells. Mol Biol Cell.

[44] Chou FS, Wang PS (2016). The Arp2/3 complex is essential at multiple stages of neural development. Neurogenesis (Austin).

[45] Kessels MM, Schwintzer L, Schlobinski D, Qualmann B (2011). Controlling actin cytoskeletal organization and dynamics during neuronal morphogenesis. Eur J Cell Biol.

[46] Zhang SX, Duan LH, He SJ, Zhuang GF, Yu X (2017). Phosphatidylinositol 3,4-bisphosphate regulates neurite initiation and dendrite morphogenesis via actin aggregation. Cell Res.

[47] Zou W, Dong X, Broederdorf TR, Shen A, Kramer DA, Shi R, Liang X, Miller DM, Xiang YK, Yasuda R (2018). A dendritic guidance receptor complex brings together distinct actin regulators to drive efficient F-actin assembly and branching. Dev Cell.

[48] Xu K, Zhong G, Zhuang X (2013). Actin, spectrin, and associated proteins form a periodic cytoskeletal structure in axons. Science.

[49] Escuin S, Vernay B, Savery D, Gurniak CB, Witke W, Greene ND, Copp AJ (2015). Rho-kinase-dependent actin turnover and actomyosin disassembly are necessary for mouse spinal neural tube closure. J Cell Sci.

[50] Galea GL, Cho YJ, Galea G, Mole MA, Rolo A, Savery D, Moulding D, Culshaw LH, Nikolopoulou E, Greene NDE (2017). Biomechanical coupling facilitates spinal neural tube closure in mouse embryos. Proc Natl Acad Sci USA.

