# Supplementary material for: Serum exosomal coronin 1A and dynamin 2 as neural tube defect biomarkers
Source: J Mol Med (Berl). 2022 Aug 1;100(9):1307–19. doi: 10.1007/s00109-022-02236-w (PMC9402777; doi:10.1007/s00109-022-02236-w)

Title: Serum exosomal coronin 1A and dynamin 2 as neural tube defect biomarkers
Journal: Journal of Molecular Medicine
Author: Yanfu Wang, Ling Ma, Shanshan Jia, Dan Liu, Hui Gu, Xiaowei Wei, Wei Ma, Wenting Luo, Yuzuo Bai, Weilin Wang, Zhengwei Yuan
Corresponding author: Zhengwei Yuan
Affiliation: Key Laboratory of Health Ministry for Congenital Malformation, Department of Pediatric
E-mail: yuanzw@hotmail.com

Supplementary Fig. 1-10

Supplementary Fig. 1 The typical images of spina bifida aperta (SBA) and other neural deformities. **(a)** Normal fetus at E12. **(b)** SBA fetus at E12 (The defect position marked in the yellow box). **(c)** Normal fetus at E14. **(d)** SBA fetus at E14 (The defect position marked in the yellow box). **(e)** Normal fetus at E16. **(f)** SBA fetus at E16 (The defect position marked in the yellow box). **(g)** Normal fetus at E18. **(h)** SBA fetus at E18 (The defect position marked in the yellow box). **(i)** Fetus with spina bifida occulta at E18. **(j)** Fetus with short tail at E18

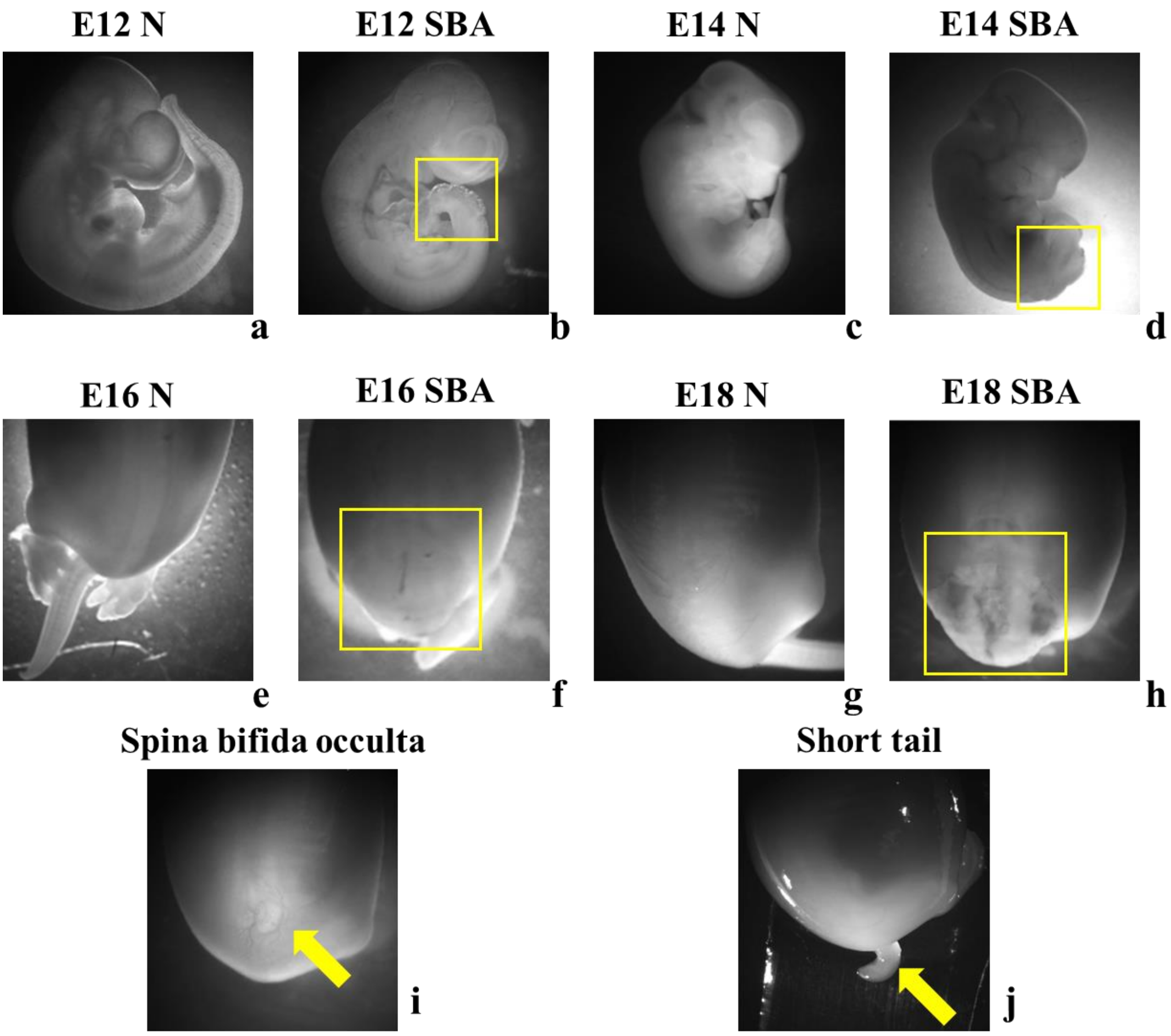

Supplementary Fig. 2 Exosome characterization. (a) Representive imaging of transmission electron microscope of exosomes isolated from serum: correct structure and size from 30-150 nm. **(b)** Dynamic light scattering of exosomes isolated from serum: adequate purity with peak means at 100±5 nm diameter. **(c)** Western blotting analysis of exosomal biomarkers (Alix, 95 kDa; CD63, 26 kDa; CD9, 24 kDa) at increasing concentration at 1, 3, and 4 μg/μL with the same trends

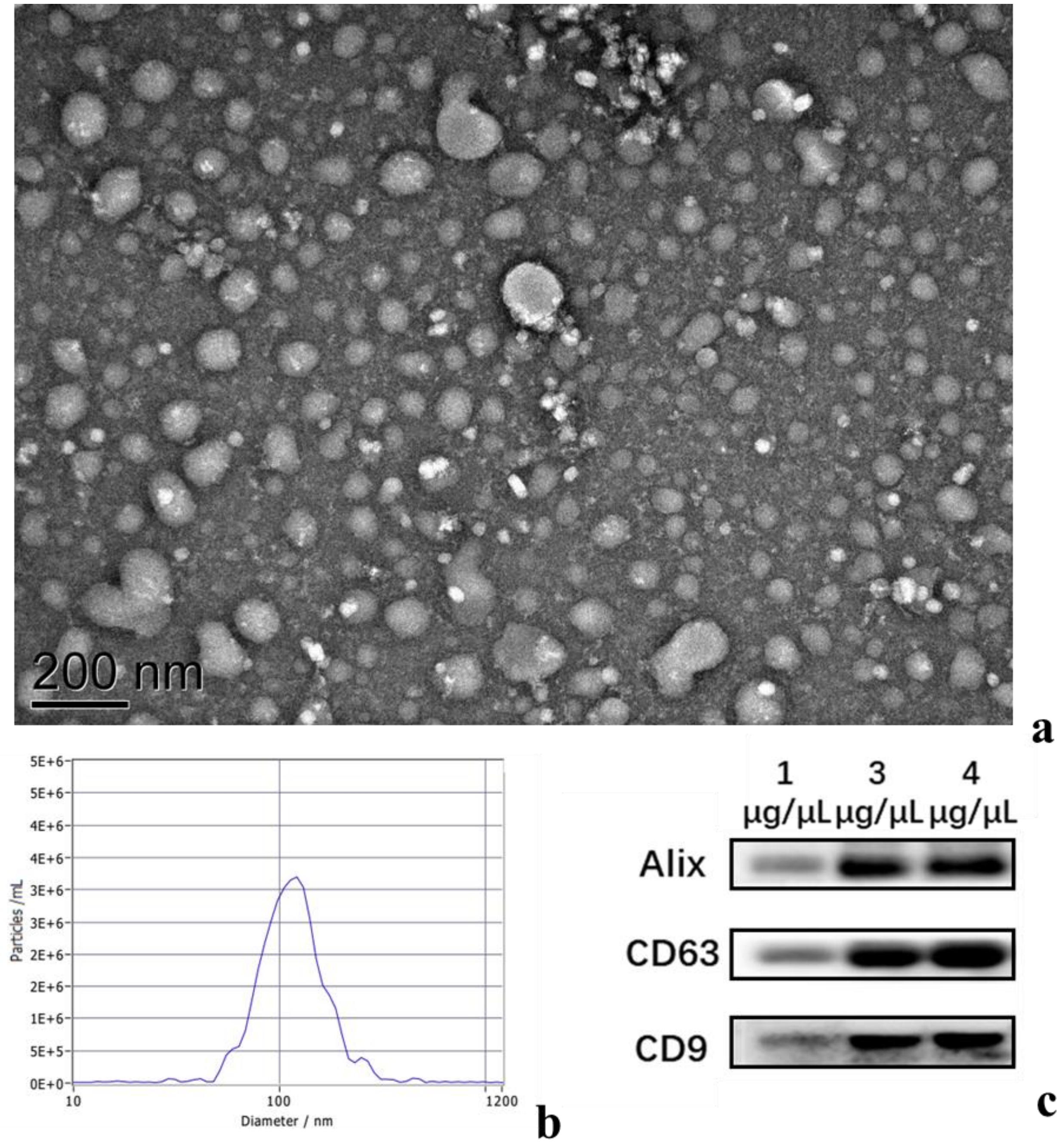

Supplementary Fig. 3 Complete western blot bands of E18 serum exosome for statistical analysis and the 4 representative bands in the main text Fig. 2a marked in the red box

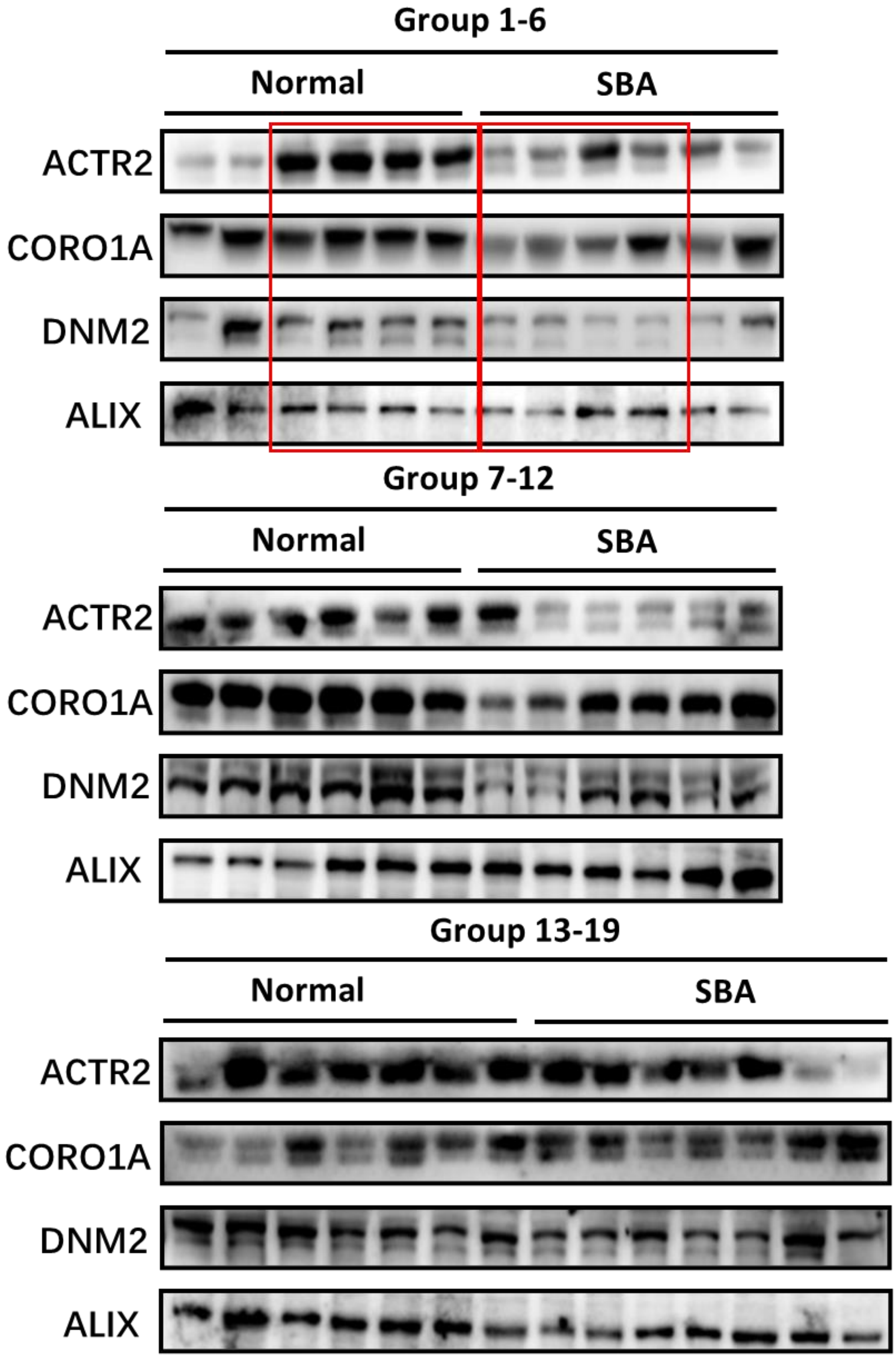

Supplementary Fig. 4 Complete western blot bands of E16 serum exosome for statistical analysis and the 4 representative bands in the main text Fig. 2b marked in the red box

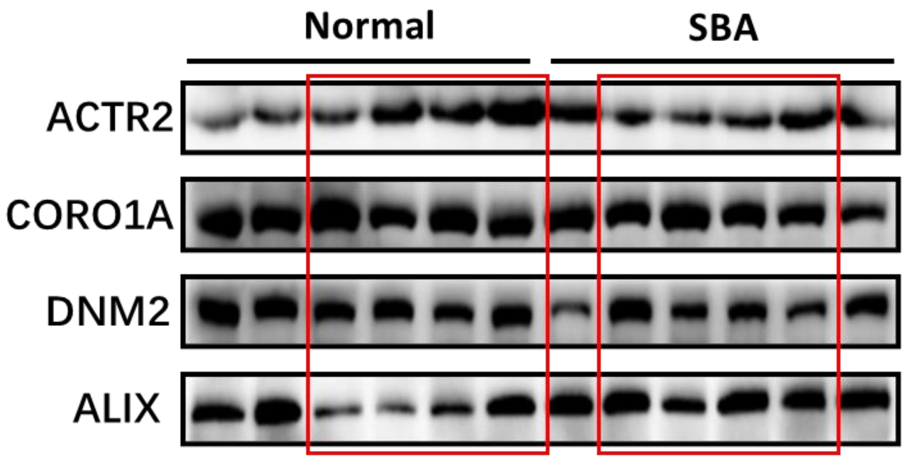

Supplementary Fig. 5 Complete western blot bands of E14 serum exosome for statistical analysis and the 4 representative bands in the main text Fig. 2c marked in the red box

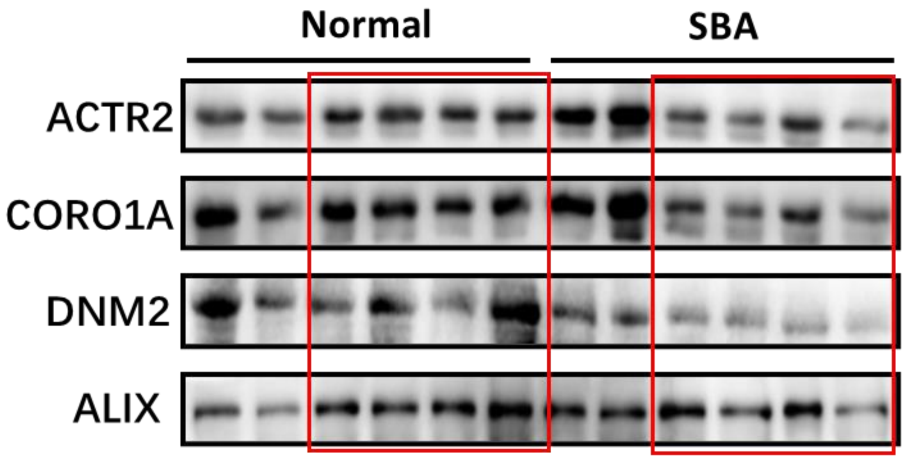

Supplementary Fig. 6 Complete western blot bands of E12 serum exosome for statistical analysis and the 4 representative bands in the main text Fig. 2d marked in the red box

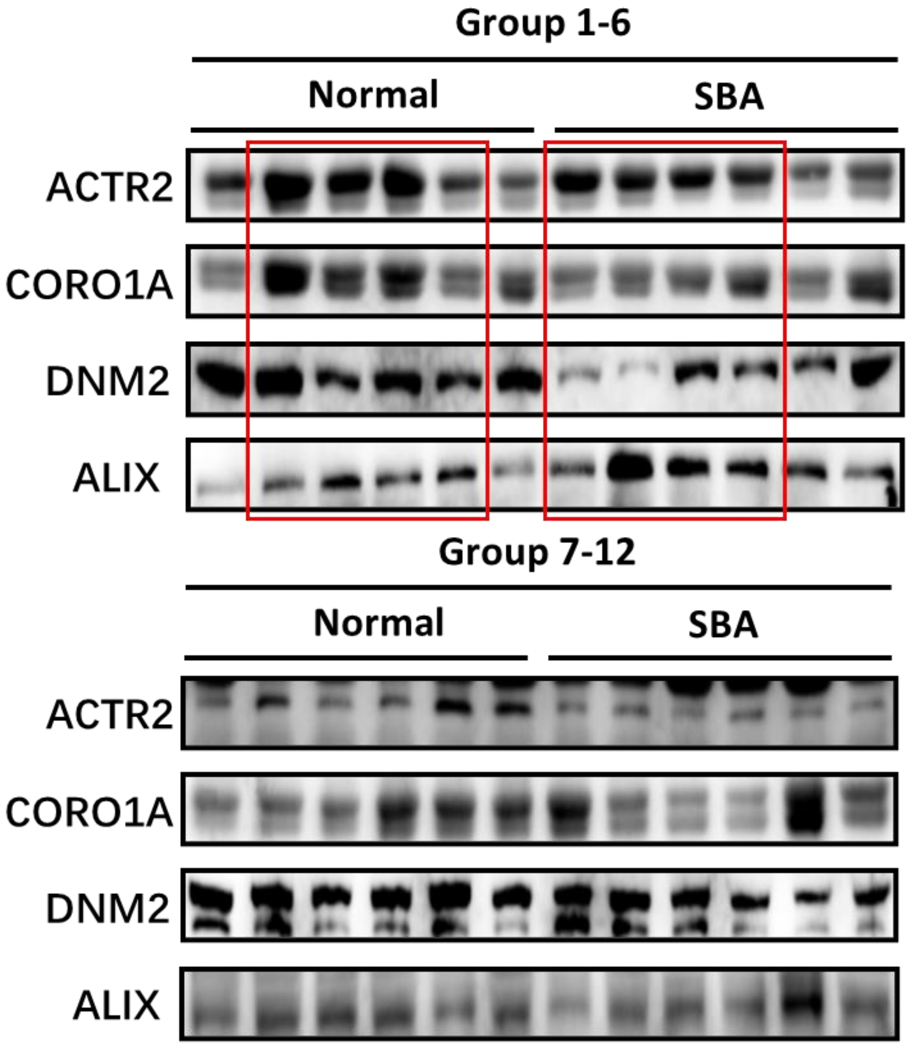

Supplementary Fig. 7 Complete western blot bands of E18 serum for statistical analysis and the 4 representative bands in the main text Fig. 2e marked in the red box

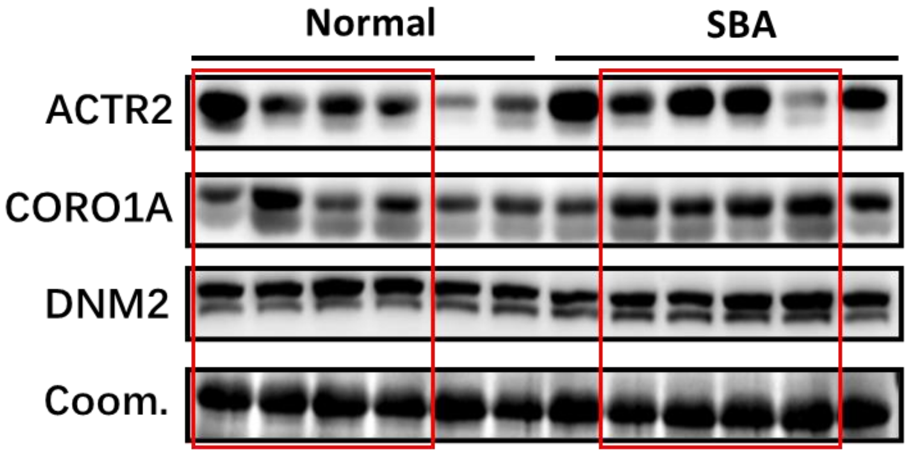

Coomassie staining of total protein of E18 serum as loading control

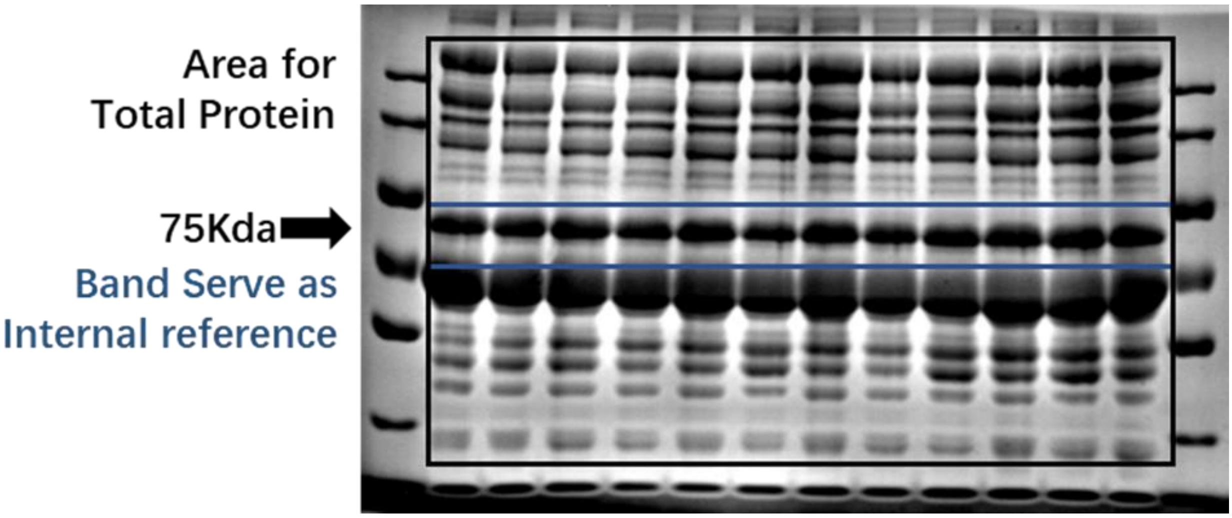

Supplementary Fig. 8 Complete western blot bands of E12 serum for statistical analysis and the 4 representative bands in the main text Fig. 2f marked in the red box

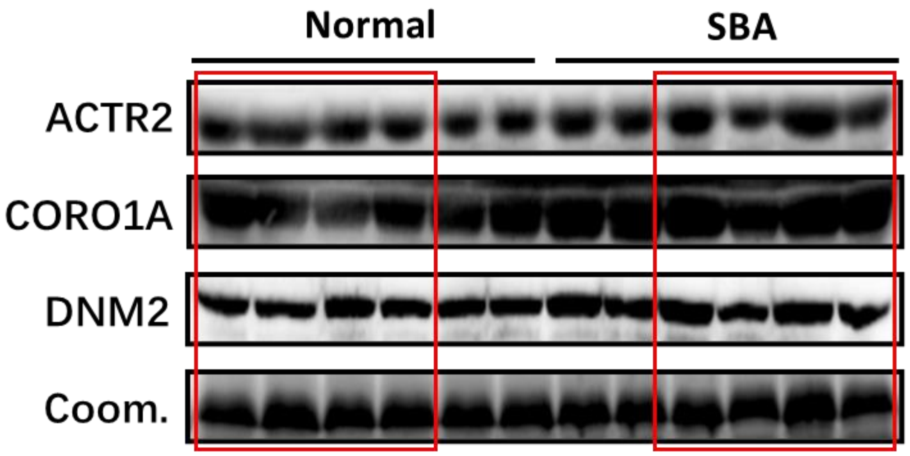

Coomassie staining of total protein of E12 serum as loading control

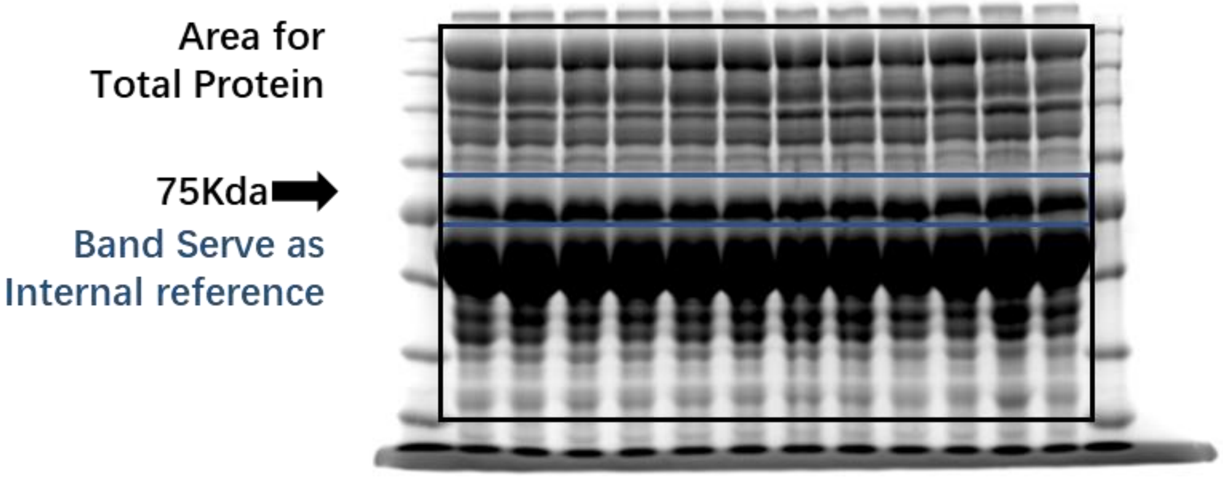

Supplementary Fig. 9 Complete western blot bands of E18 spinal cord for statistical analysis and the 4 representative bands in the main text Fig. 3a marked in the red box

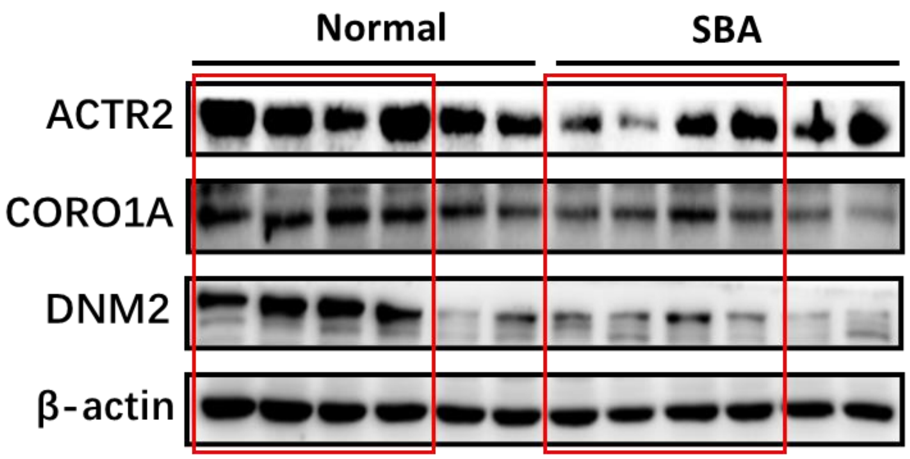

Supplementary Fig. 10 Complete western blot bands of E12 neural tube for statistical analysis and the 4 representative bands in the main text Fig. 3b marked in the red box

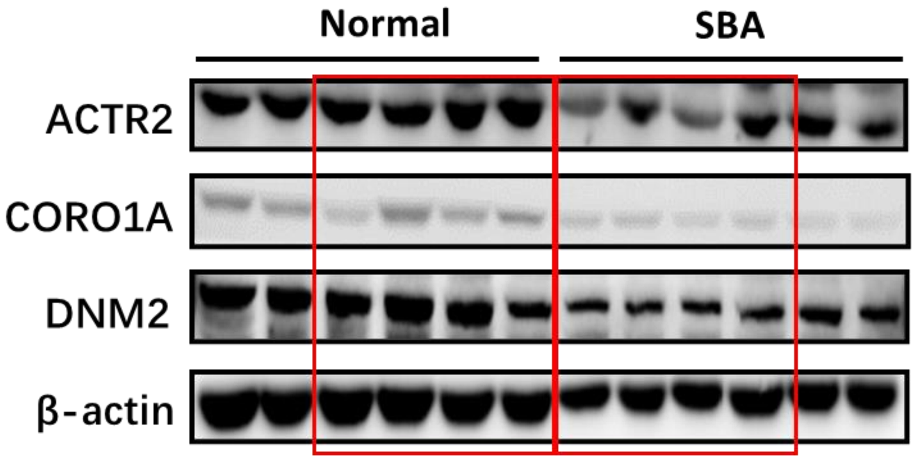

Supplement: Supplementary file 4 — Supplementary file4 (PDF 844 KB) [file 109_2022_2236_MOESM4_ESM.pdf]
